# Remote ischaemic conditioning and remodelling following myocardial infarction: current evidence and future perspectives

**DOI:** 10.1007/s10741-016-9560-9

**Published:** 2016-05-13

**Authors:** A. P. Vanezis, G. C. Rodrigo, I. B. Squire, N. J. Samani

**Affiliations:** Department of Cardiovascular Sciences, Glenfield General Hospital, University of Leicester, Leicester, LE3 9QP UK

**Keywords:** Cardioprotection, Heart failure, Remodelling, Myocardial infarction, Remote ischaemic conditioning

## Abstract

Remote ischaemic conditioning (rIC) has demonstrated its effectiveness as a powerful cardioprotective tool in number of preclinical and limited clinical settings. More recently, ischaemic *postconditioning* given after an ischaemic event such as a myocardial infarction (MI) has shown not only to reduce infarct size but also to have beneficial effects on acute remodelling post-MI and to reduce the burden of heart failure and other detrimental outcomes. Building on this platform, *repeated* rIC over a number of days has the potential to augment the protective process even further. This review considers the current evidence base from which the concept of rIC in the setting of post-MI remodelling has grown. It also discusses the ongoing and planned clinical trials which are attempting to elucidate whether the protection imparted by rIC in the preclinical setting can be translated to the clinic and become a realistic weapon in the clinician’s armoury to tackle acute remodelling and heart failure post-MI.

## Introduction

Remote ischaemic conditioning (rIC) is a non-invasive therapeutic technique whereby intermittent interruption of blood to an organ or muscle confers protection against ischaemia/reperfusion (I/R) injury to a distant organ. RIC can be implemented prior to an expected ischaemic insult (preconditioning), during the evolution of an ischaemic insult (per-conditioning) or soon after the completion of an ischaemic insult (postconditioning). For the purposes of this review, the term rIC will encompass all of these techniques.

The technique evolved from the phenomenon of local ischaemic conditioning of the heart and has been successfully used to reduce myocardial damage and improve cardiovascular outcomes in the context of primary percutaneous intervention (PPCI) for acute myocardial infarction (MI) [1, 2], elective coronary angioplasty [3–5], coronary artery bypass surgery [6], valve surgery [7] and paediatric cardiac surgery [8]. Beyond the well-established acute protective phase, early preclinical studies have hinted at an additional role for rIC, predominantly in positively influencing post-MI ventricular remodelling. In addition to directly affecting final infarct size, rIC may act to increase recruitment of stunned myocardium as well as modulating remodelling processes such as cell death with an increased emphasis on autophagy, cardiomyocyte hypertrophy, extracellular matrix (ECM) changes and the influx of pro-inflammatory cells to the damaged myocardium. This potential new role for rIC may have a profound effect in reducing the incidence and impact of post-MI heart failure.

## Remodelling following myocardial infarction

Heart failure is a major cause of long-term mortality and morbidity after MI. Analysis of registries and of large clinical trials across the western world, conducted in the era of acute revascularisation, has reported incidence rates of post-MI heart failure ranging from 10 to 50 %, depending on a number of factors including the degree and location of infarcted myocardium, how MI and heart failure were defined, whether there was pre-existing heart failure, the treatment modalities used and the characteristics of the populations analysed [9]. A retrospective analysis of Framingham Heart Study participants demonstrated an increase in the incidence of post-MI heart failure from the 1970s to the 1990s, closely linked to a decrease in mortality in acute MI, likely due to advances in myocardial salvage over this time period [10].

The development of chronic heart failure following MI most commonly results from adverse remodelling of the left ventricle, a process of structural reorganisation which occurs within the first few weeks to months after the acute event. Such remodelling is directly related to the extent of myocardial damage (due to initial necrosis and secondary apoptosis) and is most likely to occur following transmural infarction, as well as being heavily influenced by concomitant microvascular obstruction and lethal reperfusion injury in the era of acute revascularisation [11, 12]. The process of remodelling is triggered by the initial ischaemia/reperfusion insult which sets into motion a number of events. In the initial stages, the changes in the left ventricle are predominantly due to the effects of infarct expansion causing cardiomyocyte necrosis and apoptosis which ultimately leads to myocardial wall dilatation via a number of mechanisms including changes in excitation–contraction coupling and an increased expression of foetal genes leading to an alteration in proteins produced. In the later stages, remodelling is largely fuelled by hypertrophy of surviving cardiomyocytes in response to pressure and volume changes and neurohumoral signalling, reorganisation of the ECM with deposition of scar tissue and an inflammatory-driven process whereby substantial ECM turnover in border areas leads to cell slippage and further dilatation. From a whole-organ perspective, these changes impact on cardiac dimensions and function. These initial changes act to maintain an adequate cardiac output in the face of a loss of functioning myocardium; however, over time remodelling becomes maladaptive. Indeed the extent and the nature of remodelling (both compensatory and subsequently maladaptive), and its progression is a powerful predictor for both heart failure and death following MI, as well as having prognostic implications for further MI, stroke and cardiac arrest [13, 14]. Preventing or modifying some or all of the drivers for remodelling may go some way to reducing major adverse cardiovascular events in this setting.

## Local ischaemic conditioning

In 1986, Murry et al. [15] first described an endogenous cardioprotective mechanism in a canine model of MI termed ischaemic preconditioning (IPC), whereby intermittent, non-lethal occlusion and reperfusion of the left anterior descending artery (LAD) immediately prior to a period of sustained occlusion significantly reduced the final infarct size. The first in vivo study in humans assessing the effect of preconditioning was performed by Deutsch et al. [16]. In a small group of patients undergoing elective PCI for an obstructed (LAD), they showed a reduction in electrographic, metabolic and clinical markers of ischaemia during the second cycle of balloon inflation compared to the first. Yellon et al. [17] later utilised IPC prior to coronary artery bypass grafting surgery (CABG), demonstrating preserved levels of myocardial adenosine triphosphate during cardiopulmonary bypass. Over the intervening years, evolution of this technique has seen it applied to situations of unpredictable cardiac ischaemia (as opposed to anticipated ischaemia from elective surgery). Zhao et al. [18] in 2003 introduced the concept of ischaemic postconditioning (IPostC) whereby the conditioning stimulus is applied immediately or soon after the index ischaemic event by intermittent inflations and deflations of the intracoronary balloon to stagger reperfusion. Using a canine model, they demonstrated the effectiveness of IPostC in the context of acute MI with comparable levels in infract size reduction and levels of tissue oedema as well as a variety of markers of cardiac damage when compared to IPC. The possible clinical applicability of IPostC in the setting of acute coronary events was quickly realised. By staggering reperfusion during PCI by repetitively inflating and deflating the angioplasty balloon in the culprit vessel for short periods of time, Laskey et al. [19] showed a reduction in final electrocardiographic ST-segment elevation size and an increase in distal myocardial perfusion. Staat et al. [20], using a similar technique at the time of PCI, showed a significant reduction in creatine kinase release and an increase in myocardial reperfusion in the conditioned group.

## Windows of protection and delayed conditioning

Two distinct phases of cardioprotection resulting from ischaemic preconditioning have been shown to exist and are commonly termed ‘windows of protection’ [21]. The first window begins immediately following the conditioning stimulus and lasts up to 4 h. Protection within this time period is mainly induced through posttranslational modification of proteins. The second or delayed window of protection occurs 12–72 h after the conditioning event and confers protection mainly through gene transcriptional changes [22–24].

In the context of protection against the long-term effects of I/R and subsequent remodelling, the timing of the conditioning stimulus is paramount. Early studies suggested that to impart meaningful protection, conditioning must be implemented before, during or immediately after the clinical event as reperfusion injury is thought to occur within the first 15 min after the event. Dispelling this belief somewhat, Roubille et al. [25] described the damage associated with reperfusion as a ‘wave front’ and showed that rIC after I/R can be effective up to 30 min post-MI. Basalay et al. [26] also found a similar but more modest phenomenon in a rat model of I/R where rIC was effective in reducing injury when started up to 10 min into reperfusion time. The ability to impart protection, even after a significant time after the acute event, may prove clinically useful in the context of protection against adverse remodelling in post-MI in patients presenting late to hospital, as the remodelling process continues to evolve for several days after the initial insult.

## Proposed mechanisms of remote ischaemic conditioning

RIC took the concept of IPC a step further, allowing the conditioning stimulus to be applied away from the heart in a distant tissue bed. Przyklenk et al. [27] were the first to demonstrate rIC in an animal model of ischaemia/reperfusion. By preconditioning the left circumflex coronary (LCx) artery in dogs, they were able to protect the remote myocardium supplied by the LAD following transient ligation to induce MI and reperfusion. Kerendi et al. [28] later demonstrated the cardioprotective effects of rIC in the post-MI setting. After 30 min of coronary artery occlusion in rat hearts, they remotely conditioned the kidneys, then reperfused the heart and showed a 50 % decrease in infarct size compared to the control.

In humans, the most practical application of rIC is by sequentially inflating a blood pressure cuff on the arm or leg, commonly using 3–4 cycles of inflation and deflation. This non-invasive technique affords protection not only to the heart but also to a number of other organs, most notably the brain and kidneys (for review, see Ref. [29]). Although the exact mechanisms of signal transduction from the tissue/organ undergoing rIC to the target organ have yet to be elucidated, various authors have highlighted the importance of humoral and neural signalling pathways as well as modulation of the systemic inflammatory response, perhaps working in an interdependent manner [30, 31].

The humoral signalling theory postulates that blood-borne factors are released locally by the tissue undergoing rIC and are then relayed in the blood to the target organ, where they bind to G-protein-coupled receptors triggering a number of intracellular signalling pathways. A number of research groups have illustrated the importance of humoral signalling by isolating naïve animal hearts and treating them with superfusate from rIC-treated animals or human donors and demonstrating cardioprotection [32, 33]. We have shown this in our laboratory using isolated adult rat cardiomyocytes [34]. Over the years, numerous humoral factors have been implicated including adenosine, bradykinin, nitrate/nitrites, opioid peptides, prostaglandins, natriuretic peptides, endocannabinoids, angiotensin I and calcitonin gene-related peptide. It is currently believed that the signalling factor(s) is between 3.5 and 15 kDa in size and is hydrophobic [35, 36]. More recent candidates for the responsible humoral messenger include stromal cell-derived factor-1 (SDF-1a) which recruits stem cells and is activated by hypoxia [37], circulating extracellular vesicles [38] and a panel of anti-inflammatory proteins including haptoglobin and transthyretin [39].

The first evidence for the involvement of neural signalling in rIC was given by Gho et al. [40]. By administering intravenous hexamethonium (a ganglion blocker), they abolished protection afforded by remote ischaemic *pre*conditioning of anterior mesenteric artery or renal artery against sustained MI. Subsequent experiments by Ding et al. [41] showed that by directly severing the renal nerve, one could abolish the cardioprotective effect of renal ischaemia rIC in rabbits. Mastitskaya et al. [42] proposed that rIC involves transmission via vagal preganglionic neurones, whilst further studies have advocated C-fibres as the sensory neural mechanism responsible for rIC [43]. Indeed there is some suggestion that a combined humoral/neural signalling relay exists where adenosine (or other candidate factors) acts via modulation of afferent neural pathway [44]. Jensen et al. [45] demonstrated that the dialysates from type 2 diabetic individuals with peripheral neuropathy did not afford protection against infarction in a rabbit model, whereas the dialysate from non-diabetics and diabetics without peripheral neuropathy did, implying a fundamental role for neuronal signalling in this process. Furthermore, Basalay et al. [26] suggested that rIC in the pre-, per- and immediate post-MI period is heavily dependent on sympathetic messaging, whereas delayed remote ischaemic postconditioning i.e. >10 min after the event, appears not to rely so heavily on this neural signalling. This suggests a greater level of importance for humoral signalling in late postconditioning as well as potentially for repeated rIC.

A final hypothesised mechanism of rIC signalling involves modulation of the inflammatory response, important in initiating and controlling wound healing. Cheung et al. [8] demonstrated that patients given a rIC stimulus prior to undergoing open-heart surgery had a reduced systemic inflammatory response and reduced levels of cardiac damage. Li et al. [46] also highlighted the importance of inflammation by demonstrating a blunted cardioprotective response in mice deficient in NFκB (a transcription factor involved in most inflammatory processes) subjected to rIC. The importance of NFκB was underlined by Wei et al. [47] in a rat model of repeated rIC and MI where they demonstrated significantly reduction in phosphorylation of the NFκB subunit p65 and its inhibitory protein IκBα. In addition, this study showed a reduction in the infiltration of macrophages and neutrophils into the infarcted tissue in the rIC groups as well as a reduction in monocyte chemotactic protein 1 (MCP-1) in the border zone of infarcted tissue. More recently, Cai et al. [48] have shown up-regulation of expression of interleukin-10 (a potent anti-inflammatory cytokine) in a mouse model of rIC which leads to a reduction in myocardial infarct size and improved cardiac contractility.

Although some mystery still exists as to the mechanisms of rIC signalling, once the signal reaches the intended organ, the downstream intracellular pathways of rIC are thought to share much in common with local ischaemic conditioning. A number of intracellular pathways have been implicated including the reperfusion injury signalling kinase (RISK) pathway, involving ERK 1/2, p38 MAPK, PI3K-AKT and GSK3β, acting ultimately to prevent opening of the mitochondrial permeability transition pore (mPTP) at the time of reperfusion. Another important downstream pathway is the survivor activating factor enhancement (SAFE) pathway, involving activation of the JAK-STAT3/5 axis, a protective transcription factor in the context of acute ischaemia (for a detailed review, see Ref. [49]). The first window of protection is thought to depend heavily on the RISK pathway, nitric oxide (NOS), PKCε, PKCγ and reactive oxygen species. The second window of protection is more dependent on the SAFE pathway and inducible nitric oxide (iNOS) as well as retaining a significant overlap with some of the pathways implicated in the first window of protection [21, 50]. For a detailed discussion of our current understanding of the mechanisms of rIC, see the proceedings from the most recent Biennial Hatter Cardiovascular Institute Workshop [51].

## Remote ischaemic conditioning and acute myocardial infarction

The simple and safe technique of inducing ischaemia by inflating a blood pressure cuff applied to the forearm to a level greater than the systolic blood pressure was first used in the setting of acute MI by Bøtker et al. [1] in the CONDI trial. In this landmark study 4 × 5-min cycles of blood pressure cuff inflation/deflation were applied to the forearm of a cohort of ST-segment elevation MI (STEMI) patients in the ambulance on-route to PPCI and showed that with large anterior MIs caused by total occlusion of the LAD, conditioned patients had a significantly better myocardial salvage index as assessed by gated single-photon emission CT (SPECT) than the control group. A smaller study by Rentoukas et al. [2] was undertaken in STEMI patients where rIC was applied just after PCI using 4 × 4-min cycles of a forearm blood pressure cuff inflation/deflation in combination with morphine. There was a significant reduction in troponin *T* levels in the conditioned group compared to the control group as well as ST-segment deviation resolution. More recent work by White and colleagues further demonstrated the benefits of rIC, implemented in this setting just prior to PPCI in the context of STEMI. They showed a reduction in myocardial oedema and infarct size as measured by cardiac magnetic resonance imaging (cMRI) as well as reduced levels of troponins in the conditioned group [52]. The excitement generated by these trials must be tempered by the difficulty in interpreting individual studies with small sample sizes and significant population heterogeneity which often assesses non-clinical outcome measures. Reassuringly, a recent comprehensive systematic review and meta-analysis of the available trial data by Le Page et al. [53] showed significant reductions in the hard end points of MACCE and all-cause mortality in conditioned groups compared to controls in this setting.

## Remote ischaemic conditioning and remodelling postmyocardial infarction

Thibault et al. first hinted at the prospect that the effects of local IPostC after an MI may have a positive influence on myocardial contractility [54]. They demonstrated a 7 % greater left ventricular ejection fraction (LVEF) after 1 year compared with the control group (*p* = 0.04) [55]. Similarly, Munk et al. [54] in a sub-study of the CONDI trial showed that in MI patients with an area at risk (AAR) of over 35 %, those who received rIC immediately prior to PPCI had significant improvement in LVEF after 30 days compared to the control group (51 ± 11 vs. 46 ± 9 %, *p* = 0.03). Furthermore, Hoole et al. [5], as well as demonstrating reduced levels of Troponin *T* in patients undergoing elective PCI who received rIC compared to control, showed that at 6 months, the major adverse cardiac and cerebral event rate (MACCE) was lower in the rIC group (4 vs. 13 events, *p* = 0.018). More recent data published by the CONDI investigators underlined some of the long-term benefits of rIC [56]. They followed 256 patients who had suffered a STEMI to a median of 3.8 years, split equally between those who had received rIC at the time of PPCI and those who had received PPCI only. MACCE occurred in 13.5 % of the intervention group compared to 25.6 % of the control group (HR 0.49, CI 0.27–0.89, *p* = 0.018). However, due to the small sample size, no solid inferences could be made about a number of secondary outcome measures, including the development of chronic heart failure.

In all these studies, one-off rIC at or around the time of MI has pointed towards the potential for this technique to reduce the incidence chronic heart failure. However, the degree to which the difference in LVEF and other markers of heart failure is due to remodelling, as opposed to attenuation of infarct size around the time of the acute event, is difficult to ascertain. Animal studies by Reddington’s group have hinted that the progression to heart failure can be strongly attenuated, in a ‘dose-dependent manner’, by serial bouts of rIC soon after an ischaemic event. In a rat model of acute MI, Wei et al. [47] demonstrated the greatest improvement in LV chamber size, LV function and haemodynamic changes post-MI in the group that received repeated remote conditioning every day for 28 days compared to a control group and two groups receiving one-off applications of rIC either before or during ischaemia. The benefit appears to be in addition to the initial improvement seen due to reduction in infarct size and points towards novel mechanism of cardioprotection acting directly on remodelling. The study highlighted a variety of ways in which repeated rIC may work in this context including a reduction in oxidative stress, attenuation of the expression of genes associated with fibrosis and hypertrophy, and blunting of the inflammatory response with reduced levels of neutrophil and macrophage infiltration in the myocardium and reduced cytokine signalling. Previously, the same group had demonstrated that repetitive rIC significantly altered the behaviour of neutrophils after MI with reduced levels of adhesion at days 1 and 10 as well as a reduction in phagocytosis at day 10, apoptosis at days 1 and 10 and an overall change in the prolife of cytokine release [57]. More recent work from this group has suggested the existence of separate and very distinct mechanisms by which ‘one-off’ traditional rIC and repeated rIC infer protection. Whilst traditional rIC acts through the pathways described previously, repeated rIC was shown in this study to increased production of the autophagosome proteins LC3-II, cathepsin-L and Atg5 [58]. Yamaguchi et al. reinforced the power of repeated rIC post-MI and implicated exosomes as the mediators for signalling in rIC, possibly by their action of transferring anti-fibrotic microRNAs such as miR29a as well as IGF-1, which is known to be protective in the context of remodelling [59]. In addition, work by our laboratory showed that superfusate taken from ischaemic-conditioned Langendorff perfused rat hearts as well as serum taken from human volunteers immediately after undergoing rIC stimulation both independently inhibited endothelin-1-induced hypertrophy in a cellular model of hypertrophy alluding to a humoral mechanism of action [60].

## Future perspectives

Multiple studies are underway to assess the impact of one-off rIC protocols at the time of MI on various heart failure-related outcome. Following on from the first CONDI study [56], CONDI-2 (Effect of RIC on Clinical Outcomes in STEMI Patients Undergoing PPCI) is well underway. This study aims to recruit 2300 participants over a 36-months period from a number of sites across Europe (http://www.clinicaltrials.gov/ct2/show/NCT01857414) with the primary outcome of assessing cardiovascular mortality and hospitalisation for heart failure at 1 year. Completion of the study is expected in late 2016. Running in collaboration with the CONDI-2 trial is the ERIC-PPCI (Effect of Remote Ischaemic Conditioning on clinical outcomes in ST-segment elevation myocardial infarction patients undergoing Primary Percutaneous Coronary Intervention) trial. This trial has recently started recruitment and aims to recruit 2000 participants in total across multiple sites to assess whether rIC at the time of PPCI for STEMI can reduce the combined primary outcome of cardiac death and hospitalisation for heart failure at 12 months (https://clinicaltrials.gov/ct2/show/NCT02342522).

DANAMI-3 (DANish Study of Optimal Acute Treatment of Patients with ST-elevation Myocardial Infarction) aims to assess the effect of local ischaemic conditioning on heart failure rates up to 3 years following PPCI for STEMI (http://clinicaltrials.gov/show/NCT01435408). The study has completed recruitment of over 2000 participants, and preliminary results pertaining to acute outcomes have previously been presented [61]. RECOND (Reduction in Infarct Size by Remote Per-postconditioning in Patients With ST-elevation Myocardial Infarction), a Swedish-led study, aims to recruit 120 participants and apply remote *per*-conditioning during PPCI for STEMI. One of the aims of the study is to compare cMRI-assessed remodelling parameters after 180 days between the conditioned and sham groups (https://clinicaltrials.gov/ct2/show/NCT02021760). Finally, the RIC-STEMI trial (Remote Ischaemic Conditioning in ST-elevation Myocardial Infarction as Adjuvant to Primary Angioplasty) is a Portuguese-led study aiming to recruit 492 participants. Similarly, this study will recruit from patients suffering STEMI and undergoing PPCI with a 1:1 randomisation to rIC approximately 10 min prior to first angiographic balloon inflation or sham conditioning. Rather than cMRI-based outcomes, the primary endpoint in this study will be death or hospitalisation from heart failure at a minimum of 1 year (https://clinicaltrials.gov/ct2/show/NCT02313961).

Two phase II trials are underway with the hypothesis that chronic, repeated rIC use in the post-STEMI period can positively influence cardiac remodelling and reduce the incidence of and progression to heart failure: DREAM (Daily REmote Conditioning in Acute Myocardial Infarction) (http://clinicaltrials.gov/show/NCT01664611) and CRIC (Chronic Remote Ischaemic Conditioning to Modify Post-MI Remodelling) (http://clinicaltrials.gov/show/NCT01817114). The DREAM study is a UK-based, multi-centre randomised control trial recruiting individuals who have suffered a STEMI and have had successful PPCI. Inclusion criteria includes post-STEMI LVEF <45 % on transthoracic echocardiography with no prior history of MI. The study aims to recruit 72 patients and is powered to detect a 5 % increase in LVEF above natural recovery. Primary outcome data are obtained from baseline and 4-month cMRI to assess LVEF, left ventricular end diastolic volume and systolic volume, infarct size and oedema. An important facet of this trial is the intention to try and elucidate further our understanding of how much rIC in this context acts independently on remodelling when influences on the initial infarct size and MVO attenuation are reduced. This is done by beginning rIC 3 days after the acute event to avoid influencing the size of the infarct. RIC will continue for 4 weeks, performed daily by the participant. The study will randomise participants 50:50 in the intervention or the control group. The intervention group will receive a device that inflates to 200 mmHg in 4 × 5-min cycles of inflation and deflation. The control group will receive identical-looking devices that cycle as the intervention group but only inflate to a maximum of 10 mmHg. As conditioning commences on day 3 post-MI, a greater focus is on the modulation of the remodelling process rather than the infarct sparing properties of rIC.

In a similar vein, the CRIC study is a multi-centre randomised controlled trial recruiting from a STEMI/PPCI population in Canada with a recruitment aim of 82. CRIC differs from DREAM in that the investigators will recruit left anterior descending (LAD) territory infarcts only and will exclude diabetic individuals. The reasons for focusing on non-diabetic patients who have suffered large anterior STEMIs in the CRIC study are based on prior work, suggesting that this group is most likely to respond to rIC and hence gains greater impact from the intervention [1, 62, 63]. Furthermore, rIC will start just prior to PPCI and continue for 4 weeks; therefore, rIC in this context will likely have an influence on infarct size and MVO as well as subsequent remodelling. Primary outcome will be obtained by comparing cMRI at baseline and 28 days, primarily to compare LVEDV. Both the DREAM and CRIC trials are nearing completion, and it is hoped that once these trials are completed we will be in a better position to assess the role of chronic rIC in remodelling and whether this technique merits investigation with larger phase III randomised control trials.

## Challenges of remote ischaemic conditioning

The recent high-profile ERICCA trial, which showed no clinical outcome benefit at 1 year when using rIC compared to sham conditioning during elective on-pump CABG surgery, has tempered the enthusiasm in some quarters for rIC as a potential new cardioprotective therapy [64]. Pertaining to cardioprotection in the context of MI and remodelling, a number of key obstacles remain in effectively translating the protection afforded by rIC in animal and early clinical trials into larger clinical trials and ultimately into routine clinical practice.

One major challenge is that of timing of rIC. Patients having an MI presenting late to centres that can administer rIC may have completed their infarct and as such will derive minimal benefit from the procedure with regard to limiting I/R injury, although they may derive benefits from remodelling [65]. Similarly, patients presenting with small infarcts or those receiving PPCI or thrombolysis very early may derive little benefit from rIC as the scope for additional cardioprotection in this setting is limited [66, 67].

Another significant challenge is that of the large comorbidities and polypharmacy that is often encountered in the MI patient population. In particular type 2 diabetes, hyperlipidaemia, obesity and hypertension have all been shown to increase the threshold required for effective rIC [68]. Conversely, a number of the medications used in the context of MI or commonly taken by this group of patients already provide a significant degree of cardioprotection, namely ace inhibitors, statins, opioids, insulin and a number of oral hypoglycaemic agents including metformin [69]. There are also a few medication that can inhibit the effects of rIC including sulfonylureas [70]. These issues muddy the waters and make trial design and subsequent clinical translation challenging.

Finally, from a practical perspective, because rIC involves the application of a device on the arm that requires a number of inflation and deflation cycles, even with the use of an automated device, this can pose logistical problems in the ambulance or the catheter laboratory during PPCI where time is of the essence and gaining arterial and venous access with the cuff in situ may pose an issue. Furthermore, in scenarios, where rIC must be administered on a regular basis by the patient to target remodelling post-MI, the authors foresee significant concordance issues which may limit the therapy in this context. The use of automated rIC devices that can be interrogated may go some way to overcomeing this issue.

## Conclusions

RIC is only now beginning to reach its translational potential with regard to protection from ischaemic/reperfusion injury. Long-term outcome data for one-off rIC at the time of MI are awaited from the CONDI-2, ERIC-PPCI, DANAMI-3, RECOND and RIC-STEMI trials to supplement promising results from smaller preliminary studies. It is yet to be established whether early preclinical data suggesting a clinically useful role for chronic, repeated rIC use in the context of post-MI remodelling will be borne out in the trial data, but it is hoped that results from both the DREAM and CRIC trials will go some way to answering this question and potentially open the door for larger clinical trials to follow.
